# Sub‐Nanometer Electron Beam Phase Patterning in 2D Materials

**DOI:** 10.1002/advs.202200702

**Published:** 2022-06-16

**Authors:** Fangyuan Zheng, Deping Guo, Lingli Huang, Lok Wing Wong, Xin Chen, Cong Wang, Yuan Cai, Ning Wang, Chun‐Sing Lee, Shu Ping Lau, Thuc Hue Ly, Wei Ji, Jiong Zhao

**Affiliations:** ^1^ Department of Applied Physics The Hong Kong Polytechnic University Kowloon 999077 Hong Kong; ^2^ China & Polytechnic University of Hong Kong Shenzhen Research Institute Shenzhen 518000 China; ^3^ Beijing Key Laboratory of Optoelectronic Functional Materials & Micro‐nano Devices Department of Physics Renmin University of China Beijing 100872 China; ^4^ Department of Chemistry and Center of Super‐Diamond & Advanced Films (COSDAF) City University of Hong Kong Kowloon 999077 Hong Kong; ^5^ China & City University of Hong Kong Shenzhen Research Institute Shenzhen 518000 China; ^6^ Department of Physics Hong Kong University of Science and Technology Clear water bay Hong Kong 999077 China

**Keywords:** 2D materials, electrical contact, phase patterning, scanning transmission electron microscopy (STEM), sub‐nanometer

## Abstract

Phase patterning in polymorphic two‐dimensional (2D) materials offers diverse properties that extend beyond what their pristine structures can achieve. If precisely controllable, phase transitions can bring exciting new applications for nanometer‐scale devices and ultra‐large‐scale integrations. Here, the focused electron beam is capable of triggering the phase transition from the semiconducting T’’ phase to metallic T’ and T phases in 2D rhenium disulfide (ReS_2_) and rhenium diselenide (ReSe_2_) monolayers, rendering ultra‐precise phase patterning technique even in sub‐nanometer scale is found. Based on knock‐on effects and strain analysis, the phase transition mechanism on the created atomic vacancies and the introduced substantial in‐plane compressive strain in 2D layers are clarified. This in situ high‐resolution scanning transmission electron microscopy (STEM) and in situ electrical characterizations agree well with the density functional theory (DFT) calculation results for the atomic structures, electronic properties, and phase transition mechanisms. Grain boundary engineering and electrical contact engineering in 2D are thus developed based on this patterning technique. The patterning method exhibits great potential in ultra‐precise electron beam lithography as a scalable top‐down manufacturing method for future atomic‐scale devices.

## Graphical abstract

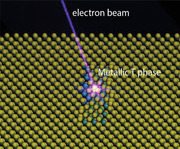

## 1 Introduction

The discovery of metal‐semiconductor phase transitions offers new opportunities in applications, including intelligent computing,^[^ ^1^ ^]^ sensor,^[^ ^2^ ^]^ and memory^[^ ^3^ ^]^ devices. However, continuous scaling‐down of devices currently requests the capability of phase control on materials with atomic, at least few‐nanometer‐scale precision, which cannot be achieved with mechanical,^[^ ^4^ ^]^ thermal,^[^ ^5^ ^]^ chemical,^[^ ^6^ ^]^ and related regular methods. Optical patterning,^[^ ^7^ ^]^ although compatible with current photolithography technology,^[^ ^8^ ^]^ is limited by the diffraction of the using light.^[^ ^9^ ^]^ Alternatively, high energy electron beams that own much smaller beam size, wavelength, and influential area^[^ ^10^ ^]^ hold great potential in atomic‐level phase patterning. Meanwhile, patterning metallic features on semiconductors is an essential fabrication step for microelectronics. A 7 nm integrated circuit (IC) has been launched by extreme ultraviolet (EUV) photolithography with 13.5 nm wavelength,^[^ ^8^ ^]^ reaching the inherent limit set by optics and materials.^[^ ^9^ ^]^ It is certain that for patterning the atomic‐scale devices, the electron (e^–^) beam has significant advantages over photons in terms of wavelength and beam size, yet it also encounters scattering and damage problems^[^ ^10^ ^]^ that prevent single‐exposure electron beam lithography from entering the sub‐10 nm regime and further industrial applications.

**Figure 1:**
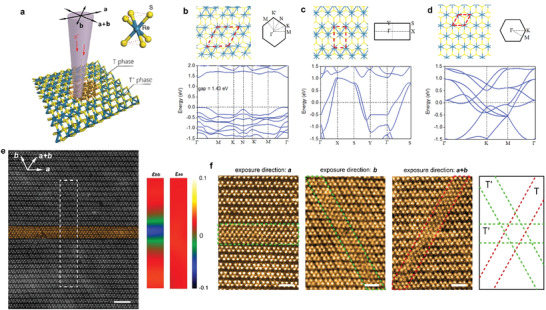
Polymorphism and one‐dimensional（1D) phase patterning in 1L ReS_2_. a) The scheme of 2D ReS_2_ phase transition under STEM. *a*,*b* and *a + b* are the three low index directions of ReS_2_. e^–^ beam exposure creates a new T phase embedded in the pristine T′ phase. b–d) Atomic structures and electronic structures of T’’ (tetramerization in two directions) phase, T’ (dimerization in one direction) phase, and T (no dimerization) phase from DFT calculation, respectively. e) STEM high‐angle annular dark‐field (HAADF) image (left) after 1D phase patterning (e^–^ beam exposure area false‐colored by orange) and strain analysis (*ε*_bb_ and *ε*_aa_) results (right) for white dashed box area in left HAADF image. Scale bar = 2 nm. f) STEM HAADF images of atomic‐scale phase transition from pristine T'' phase into 1D T’ or T phases, via 1D e^–^ beam exposure direction along ***a***, ***b*** and ***a+b*** crystal directions (scheme on the right), respectively. False color is applied to STEM images. e^–^ beam scanning areas are marked by green and red boxes. Scale bars = 1 nm.

Recently, semiconducting‐metallic polymorphic structures have been discovered in 2D materials via laser irradiation^[^ ^7^, ^11^ ^]^ and patterned electronic/chemical doping.^[^ ^6^, ^12^ ^]^ The hexagonal (H) and tetragonal (T/T’/T_d_) phases in 2D transition metal dichalcogenides (TMDs)^[^ ^13^ ^]^ prove to be ideal candidates for implementing phase engineering^[^ ^14^ ^]^ and enable various novel device structures such as high‐quality heterojunctions and ohmic contacts in 2D molybdenum disulfide (MoS_2_)^[^ ^15^ ^]^ and molybdenum ditelluride (MoTe_2_),^[^ ^7^ ^]^ respectively. Aside from extensively investigated H‐T phase transitions, phases transform between the metallic T phase and their charge density wave (CDW) phases^[^ ^16^ ^]^ (viz., T’ or T’’) (**Figure** **1**a) in 2D TMDs with zigzag, ribbon, or diamond‐shaped clusters of metal ions are less understood. Particularly, besides the prevailing T’’ phase (distorted T phase), a 20.6% of spacing shrinkage in several diamond chains under e^–^ irradiation is observed,^[^ ^17^ ^]^ which shows great promise in completing the transition into T phase and exploring related mechanisms in 2D ReS_2_ or ReSe_2_.

Here, we address the precise generation of the metastable T’ (space group: P21/m) and T (space group: P‐3m1) phases in 1L ReS_2_ and ReSe_2_^[^ ^17^, ^18^ ^]^ from the original T’’ phase (space group: P‐1) under e^–^ beam exposure. Our experimental and density functional theory (DFT) results reveal that the T and T’ phases are stabilized by in‐plane compressive strain in the ReS_2_ and ReSe_2_ monolayers with negligible out‐of‐plane strain, which is induced by the knock‐on effect^[^ ^19^ ^]^ of S or Se atoms from high energy e^–^ beam irradiation. More importantly, such phase transitions can be precisely triggered down to the Angstrom scale, and the transformed structure is controllable by irradiation conditions. Our in situ transmission electron microscopy (TEM)‐scanning tunneling microscopy (STM) reveals the superior improvement of conductivity during the phase transition process, exhibiting the designable and scalable fabrication of metallic wires and/or pads embedded in the semiconducting T’’ phase.

## 2 Results and Discussions

The theoretical atomic structures, their associated Brillouin zones, and electronic band structures of the freestanding T’’ (tetramerization in two directions), T’ (dimerization in one direction), and T (no dimerization) phases of monolayer (1L) ReS_2_ are shown in Figure 1b–d. The lattice constant of the most stable gapped T’’ phase is, even in the presence of tetramerization of Re atoms, significantly expanding by ≈16–18% than that of the metallic T phase, while the anisotropic T’ phase holds the constant value in between.

Large pristine 1L ReS_2_ is prepared by chemical vapor deposition (CVD) and confirmed as the T'' phase under STEM (Figure 1e). To trigger the phase transition in 1L ReS_2_, we primarily apply the STEM mode in our electron microscope with a converged e^–^ beam of ≈1.5 Å (see Methods). Two excitation modes are used to stimulate likely phase transitions, namely line mode and surface mode. In the line mode, e^–^ beam is sequentially exposed onto a quasi‐1D area in 1L ReS_2_ along <100> directions of the monolayer (see Methods), denoted as exposure directions *a*, *b* and *a + b* (Figure 1e,f), respectively. The e^–^ beam scanning directions in STEM images are identical to the 1D exposure directions. Figure 1e shows the result after exposing along direction *a*, same as a zoomed‐in image shown in the left panel of Figure 1f. The 1D exposure area is ≈1 nm in width, highlighted by the green and red dashed lines in Figure 1f.

Under e^–^ beam exposure, the atomic‐scale areas of 1L ReS_2_ undergo beam irradiation has evident atomic structural transformations, while the surrounding lattice structures without irradiation stay pristine. The e^–^ beams seem to be capable of collapsing the Re‐Re bonds in the di‐ or tetramerized Re clusters along the beam exposure direction (*a* or *b*), and reshaping the diamond‐shape Re_4_ clusters (T’’) into zigzag Re_2_ chains (T’) (Figure 1f, left and middle). At the same time, the 1D exposure along *a + b* direction ruptures most Re‐Re bonds in both directions, giving rise to a three‐unit‐cell‐width T phase embedded in the pristine T’’ phase (Figure 1f, right). The lattice spacings of three phases along two domain directions are measured in Figure S1 (Supporting Information), and the atomic structures of the T and T’ phases are in accordance with our DFT calculations, as elucidated later. Geometric phase analysis (GPA) on STEM images indicates a 1D strain distribution in the exposure area (Figure 1e). In all three cases, there is compressive strain perpendicular to the 1D exposure direction, while almost zero strain exists along the 1D exposure direction. The above results well demonstrate converged e^–^ beam irradiation as an effective technique in precise phase patterning on 2D ReS_2_ down to the Angstrom scale.

**Figure 2:**
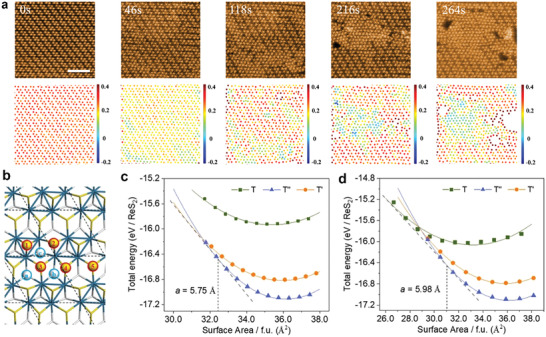
2D phase patterning in 1L ReS_2_ and calculated results on strain effects on 1L ReS_2_. a) STEM HAADF series during 2D phase patterning (up) and the corresponding von Mises dilatational strain mapping (down). Scale bar = 2 nm. b) Relaxed atomic structure of 1L T’’ ReS_2_ and different S atomic positions for knock‐on energy calculations (corresponding to Table S1, Supporting Information). The red circle marks the bottom S atom, and the blue circle marks the top S atom. c) Energy‐Surface Area (*E‐S*) relations of T, T’ and T’’ phase under the uniaxial strain along ***a*** crystal direction. Different phases are shown by different symbols: T phase, green squares; T’ phase, orange dots; T’’ phase, blue triangles. d) *E‐S* relations of T, T’, and T’’ phase under biaxial strain. Tangent lines are presented by the gray dotted lines.

Next, the phase patterning in 2D areas on 1L ReS_2_ (the surface mode) is carried out, whereas the focused e^–^ beam repeatedly scans in selected rectangular areas. **Figure** **2**a and Figure S2 (Supporting Information) exhibit continuous changes in STEM images acquired under e^–^ beam irradiation during surface mode with an exposure area of ≈20 × 20 nm, fully covering the viewing areas. Initially, the e^–^ beam mainly creates S (white triangles) atomic vacancies resulting from the knock‐on effect, accompanied by a few Re (blue triangles) vacancies (Figure S2a, Supporting Information). The atomic positions of all Re atoms in the viewing area are extracted from the STEM images for phase identification. New phases, including T and T’, other than the pristine T’’ phase, gradually emerge, as encoded in the blue color (Figure S2a, Supporting Information) for each snapshot. These phases are distinguished by measuring the Re‐Re atomic distances of the nearest neighbors (see Methods). After 100–200 s, the nuclei of the T phase start to emerge, leaving appreciable nanoscale pores in the selected area under an incremental irradiation dose (Figures 1 and 2a). The emerging process persists to ≈200 s, afterward the number of Re atoms in nuclei rapidly increases from 50 to 100 (Figure S2b, Supporting Information), shown as an estimated critical nuclei size of ≈2.5nm × 2.5nm for the T phase embedded in the T’’ phase. From atomic strain mapping, inside the T phase the strain is close to 0, the value of which is much larger on the edge of pores and junction area with T’’ phase. Hence these nanoscale pores effectively relax the strain led by the reduced lattice constant from the original T’’ phase to prevent the membrane from collapsing in a large area. Introduction of additional Re defects usually delays the 1T’’ to 1T transition (Figure S3, Supporting Information) because of the prevail twist of diamond chain directions. Meanwhile, additional S vacancies can be recognized from integrated differential phase contrast (iDPC) STEM images (Figure S4, Supporting Information) that show the concentration of S vacancies is 7.5% in the T’ and 6.2% in the T phase, much higher than ≈1% in the pristine T’’ phase in the beginning.

Results from density functional theory (DFT) calculation explain that knock‐on effects^[^ ^19^ ^]^ are the primary beam effect on monolayer samples under high energy e^–^ irradiation, which creates atomic vacancies. Formation energies of S vacancies (single‐ and bi‐ vacancies) and their associated displacement threshold energies (*T*_d_) of 1L ReS_2_ were revealed by DFT calculations and illustrated in Figure 2b and Table S1 (Supporting Information). Single S‐3 vacancy (Figure 2b) has the smallest *T*_d_ of 5.88 ( ± 1.04) eV, much lower in comparison with other single‐ and bi‐ S vacancies (>6.29 ±  1.04 eV). It indicates S‐3 vacancy is the easiest one to be created with the corresponding electron beam threshold energy (*E*_e_) of 79.7 ±  13.3 keV. In practice, the 1L ReS_2_ structure is robust to prevent the monolayer from collapsing because of the largely suppressed formation of extended defects (e.g., di‐vacancies or larger defects). Electron energy of 60 keV from our STEM is adequate to be highly selective in generating single S vacancies (mainly single S‐3 vacancy) by slow damages, which is consistent with the experimental observation in Figure S4, (Supporting Information 0 s).

Upon the generation of single S vacancies under the e^–^ beam irradiation, two major effects occur on the 1L ReS_2_ sample: n‐doping from the electronic side and in‐plane compressive strain due to bond contraction surrounding the S vacancies from the mechanical side. Our DFT calculations compare the relative stability of three phases, T, T’, and T’’, under electron doping (Figure S5, Supporting Information). Given their equilibrium lattice constants, limited influences are caused by electron doping that the order of relative stability does not change up to 0.5 e^–^ per ReS_2_, implying the compressive strain introduced by S vacancies plays a much more critical role.

2D von Mises strain is measured from serial STEM images during the phase transitions depicted in Figure 2a (see Methods). The dilatational strain invariants shown in Figure 2a can evaluate the bi‐axial normal strains in 2D samples, considering the shear strain invariants kept almost constantly at zero throughout the sample positions and phases (Figure S6, Supporting Information), the out‐of‐plane bending is excluded during phase transitions. There are also no ripple‐induced lattice distortions (Figure S7, Supporting Information) in the new T phase regions. From 0 to 46 s (left two panels of Figure 2a), as expected, a uniform 2D compressive strain is observed before the phase transition in the 1L ReS_2_ sample, which arise from single S vacancies under e^–^ beam. At 118 s (third left panel of Figure 2a), the T’’ to T phase transition starts, and non‐uniform strains emerge at several locations, strains in intact T’’ phase areas are released, and their dilatational strain invariant comes back to that of the pristine state ( $\eta_{m}^{2D}$ = 0.4). After 216 s (middle panel of Figure 2a), the 1T phase area is gradually enlarged. Experimentally, the critical dilatational strain invariant for phase transition from T’’ to T is $\eta_{m}^{2D}$ = 0.13 ($\eta_{m}^{2D}$ for the equilibrium T phase is set as 0). The uniform bi‐axial compressive strain observed in our GPA results ensures that the phase transition does not cause significant out‐of‐plane structural fluctuations; otherwise, there would be apparent inclination‐induced inhomogeneity of strain distribution and curvy distortions in STEM images (Figure S7, Supporting Information).

Therefore, the compressive strain, which results from e^–^ beam generated S vacancies, plays a major role in both 1D (Figure 1) and 2D (Figure 2) e^–^ beam patterning. The compressive strain usually originates from the bond contraction near the dangling bonds at defect positions.^[^ ^20^ ^]^ Remarkably, a uniaxial strain condition is reached in 1D exposure because of the linear removal of S atoms, and the bi‐axial compressive strain for all 2D cases is given by evenly created single S vacancies in 2D exposed zones, both of which closely associate with the type of the phase attained. Based on this point, by the electron beam exposure, the amount of S and Re vacancies can be controlled. In general, the electron dose can be adjusted by the beam current, dwell time, and pixel size, which eventually triggers the phase transition. Hence the beam current and dwell time and the scanning area eventually control the electron irradiation dose. More specifically, though the e^–^ beam exposure sometimes generates nanoscale pores in the sample, a larger area with mixed T’’/T’/T phases can be obtained with a relatively lower dose by using a lower magnification and larger scanning and exposure area (Figure S8, Supporting Information), and phase transition without nanoscale pores can be acquired with a faster scanning speed at a lower dose rate (Figure S9, Supporting Information). Due to the limit of the field of view and sample size inside the TEM, the scalability of phase patterning stays at the nanometer scale (up to 10nm × 10nm) by the current method of e^–^ beam exposure. However, the scalability can be improved by a specific design of e^–^ beam exposure system for such phase patterning later on.

Our DFT calculations reveal the energy‐surface (*E‐S*) relations of the three phases in 1L ReS_2_ under strain. The total energy of uniform T (green), T’ (orange), and T’’ (blue) phases is plotted in Figure 2c,d as a function of their surface areas with uniaxial (Figure 2c) and biaxial (Figure 2d) strains. In terms of the uniaxial case, the T’’ and T phases appear the most stable and unstable at the equilibrium of the T’’ phase, respectively. The stability superiority of the T’’ phase reduces upon a compressive strain along lattice direction *a*. A crossover of the total energies of T’’ and T’ phases is found when the surface area of the T’’ unit cell reaches 32.4 Å^2^. The tangential line connecting the *E‐S* curves of these two phases indicates a transition lattice spacing of 5.75 Å. Yet the T phase remains very unstable under uniaxial compressive strain, and it becomes the most stable phase when a biaxial strain is applied (Figure 2c), along with an estimated transition lattice constant of ≈5.98 Å. Both transition lattice constants, namely 5.75 and 5.98 Å, with the S defects neglected, are comparable with the experimentally derived lattice constants measured as 5.81 Å for T’ and 5.76 Å for the T phase that include S vacancies (Figure S10, Supporting Information).

Following the same strategy as 1L ReS_2_ patterning, we also achieve 1D and 2D e^–^ beam phase patterning on 1L ReSe_2_, as shown in Figure S11 (Supporting Information). Compared with the T’’ phase 1L ReSe_2_, the lattice constants of the T phase shrink by ≈15–18% during the experiment under the same strain‐induced phase transition mechanism. Thus, the precise e^–^ beam phase patterning approach is most likely, a general technique applicable to many 2D materials.

**Figure 3:**
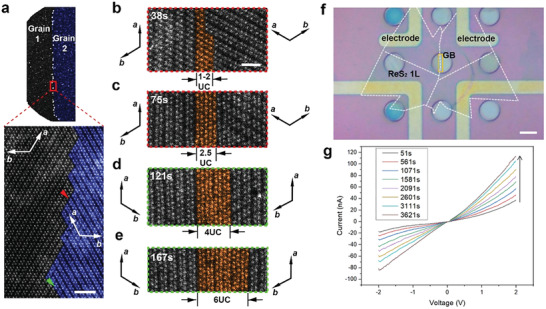
Grain boundary engineering by e^–^ beam phase patterning. a) Low magnification (MAG) and High‐MAG STEM HAADF images for pristine zigzag GB in 1L ReS_2_. *a* and *b* crystal directions are marked. Two grains are false‐colored for distinction. Scale bar = 2 nm. b,c) T phase patterning from 38–75 s corresponding to the GB section marked by the red triangle in (a). Scale bar = 1 nm. d,e) T phase patterning corresponding to the GB section marked by the green triangle in (a). The same scale is applied in (b–e). Crystal directions are marked beside the respective grains in (b–e). f) In situ TEM electrical testing on the grain boundary in 1L ReS_2_ using Protochips Fusion. Scalebar = 8 µm. g) The IV results correspond to (f) after gradual e^–^ beam phase patterning on the GB area.

Phase transitions in uniform phase domains aside, the patterning approach is also applied to grain boundaries (GB)^[^ ^21^, ^22^ ^]^ of the T’’ phase ReS_2_ monolayers. Prevailing deep energy states in the bandgaps are spatially found at GBs of the ReS_2_ monolayer,^[^ ^21^ ^]^ which induce Fermi level pinning and greatly lower the carrier mobility across GBs.^[^ ^23^ ^]^ We, thus, intend to eliminate the pinning levels by triggering the T’’ to T or T’ phase (semiconductor to metal) transition at GBs in order to improve carrier transport properties across the GBs. A typical GB in the ReS_2_ monolayer, as shown in **Figure** **3**a, is atomically sharp with zigzag sections, in which the T phase preferentially emerges between the two grains under e^–^ beam exposure in the 2D surface mode (Figure 3b–e). The e^–^ beam scanning area is ≈10 × 10 nm covering the GB area. As a result, the GB section (marked by the red triangle in Figure 3a) can be e^–^ beam patterned into T phase in 1–2 unit cell (UC) width to 2.5 UC width from 38 to 75 s (Figure 3b,c), while the GB sections marked by the green triangle in Figure 3a can be patterned into T phase as well, the width is enlarged from 4 to 6 UC from 121 to 167 s (Figure 3d,e).

Continuous e^–^ beam exposure enlarges the area of the transformed T phase on the GB, which as expected, improves the carrier transport properties across the GB after the T phase eventually replaces the original T’’ phase at the GB. We thus transfer 2D ReS_2_ flakes onto an in situ TEM chip (Protochips Fusion), shown in Figure 3f where the white dashed lines highlight the contour of the edges of a ReS_2_ monolayer flake. It shows an electrical circuit containing the 1L ReS_2_ flake with a GB in between the paired electrodes. After the focused e^–^ beam scanning on the GB area in the yellow dashed box (Figure 3f), the electrical conductance between the two electrodes gradually increases up to ten folds, as read from the current‐voltage (*I*‐*V*) responses of the paired electrodes (Figure 3g). The e^–^ beam scanning time is prolonged due to the larger scanning area in this case. Hence the e^–^ beam metallic phase patterning can serve as an efficient, precise GB engineering or even general defect engineering technique for 2D semiconductors.

**Figure 4:**
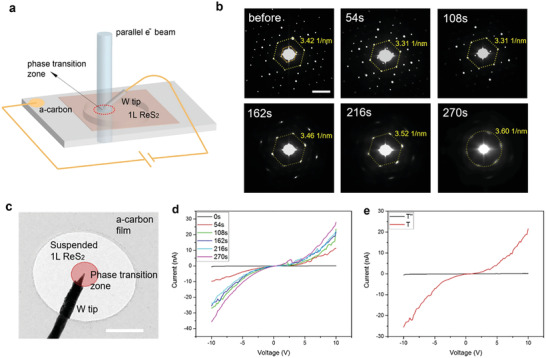
Scaling up of e^–^ beam patterning and electrical contact engineering on 1L ReS_2_. a) Scheme of parallel e^–^ beam exposure and in situ TEM‐STM setup. b) Typical evolution of selected area electron diffraction (SAED) patterns on 2D ReS_2_ during parallel e^–^ beam patterning. The lattices clearly shrink, and the reflexes for superlattices (inner hexagon) varnish after e^–^ beam exposure, showing the formation of T phase. Scale bar = 5 1 nm^−1^. c) in situ TEM image of 1L ReS_2_ contacted with a W tip and exposed by parallel e^–^ beam, red circle zone indicates the e^–^ beam exposure area. Scale bar = 0.5 *μ*m. d) *I‐V* curves with e^–^ beam exposure time (0–270 s). e) Direct comparison of *I‐V* curves on T’’ phase and T phase 1L ReS_2_.

In addition to the focused e^–^ beam exposure approaches above, we also explore the scaling up parallel beam exposure for phase patterning, as monitored by selected area electron diffraction (SAED) on a larger scale. A 200 kV electron beam in TEM mode is employed to convert the T’’ phase in a 2D area of 0.3 *μ*m^2^ into the T/T’ phase on 1L ReS_2_ (**Figure** **4**a) (Methods). The feasibility of a parallel e^–^ beam manifests the scaling‐up potential for the e^–^ beam approach. The SAED patterns are shown in Figure 4b. The anisotropic diffraction patterns gradually turn into isotropic, with the smearing of reflexes due to lattice strain variations. The (110) *g* vectors are highlighted, showing a reduction of lattice constants by 9–12% under the e^–^ beam from 54 to 270 s, in consistence with the lattice changes after the transition into T/T’ phases. This parallel e^–^ beam exposure strategy allows for higher throughput phase patterning for larger areas, complementing the high‐precision phase patterning by focused e^–^ beams.

Theoretical electronic band structures (Figure 1b–d) clearly show that the T and T’ phases of 1L ReS_2_ are both metallic, irrespective of strain conditions (Figure S12, Supporting Information). We further performed in situ TEM‐STM experiments^[^ ^24^ ^]^ to locally probe the electrical conductivities of the newly patterned T/T’ phases. During the process of parallel e^–^ beam exposure on 1L ReS_2_, an in situ tungsten STM tip is manipulated to make electrical contact with the generated T/T’ phase area, and I‐V signals were sequentially recorded (Figure 4c,d). Similar to our previous work,^[^ ^24^ ^]^ the contact between the tungsten (W) tip and the 1L‐ReS_2_ sample is ensured mechanically stable by multiple and repeatable IV testing to maintain a constant contact area. The measured conductivities, therefore, directly reflect the evolution of local conductance of the contact^[^ ^24^ ^]^ within the e^–^ beam exposed area. In comparison with typical electrical contact results for the new T phase (Figure 4d,e), the electrical conductance for the contact is significantly increased tenfold by e^–^ beam exposure for 54 s and then increased to 37‐fold after 270 s exposure. Though there are other factors, including the defect states or strains that might have an influence on the electrical contacts, the area exposed under the e^–^ beam here is much larger than the exact contact area between the STM tip and ReS_2_, and normally vacancy defects tend to lower rather than improve the electrical conductance of 2D devices. Hence we believe the total conductance gradually increases in our experiment as a larger T phase area is generated by the e^–^ beam exposure. Overall, the substantially enhanced conductance can be rationalized by the metallic nature of the T phase. Throughout the entire in situ TEM‐STM process, the integrity and stability of the 1L ReS_2_ membrane were maintained, and no crack/hole over 10 nm was observed. Thus, herein we provide a method for electrical contact engineering using e^–^ beam irradiation.

## 3 Conclusion

We demonstrate that down to atomic precision, the focused e^–^ beam patterning technique is capable of engineering the metallic T or T’ phase from 1D line to 2D surface at both grain domains and boundaries on the semiconducting T’’ phased ReS_2_ and ReSe_2_ monolayers. It provides an ideal patterning precision up to the sub‐Å scale after aberration correction^[^ ^25^ ^]^ and results in phase patterning areas from several to ≈100 nm^2^, which is orders of magnitude greater than any conventional lithography techniques. The flexible patterning conditions allow e^–^ beam energy to range from 60 to 200 kV without collapsing the materials, promoting further applications such as grain boundary engineering and electrical contact engineering at the nanometer scale. Besides, the operational speed of e^–^ beam patterning is, in principle, much faster than traditional scanning probe microscopy (SPM) techniques for atom‐by‐atom lithography or structuring.^[^ ^26^ ^]^ The e^–^ beam size and dose can easily scale up in both focused and parallel beam modes, while the parallel mode offers an even faster speed of patterning. The e^–^ beam knock‐on induced and in‐plane strain mediated metal‐semiconductor phase patterning technique provides powerful means for atomic‐scale structuring, which can fuel the future development of nanotechnology.

## 4 Experimental Section

### Synthesis of Monolayer ReS_2_ and ReSe_2_

Monolayer (1L) rhenium disulfide (ReS_2_) was grown on a single‐side polished c‐face sapphire substrate by the chemical vapor deposition (CVD) method under atmospheric pressure. Sulfur powder (Aldrich, 99.998%) was put in the upstream zone, while ammonium perrhenate (NH_4_ReO_4_) (Aldrich, 99.999%) was put in the downstream zone as precursors, with a weight ratio of 50:1 in a two‐zone splitting tube furnace. The polished side of the c‐face sapphire substrate was toward the top of the Re source, and the size was 1 cm × 1 cm. To purify the gas atmosphere of the tube before heating, 300 standard cubic centimeter per minute (sccm) Argon (Ar) gas was purged for 10 min. Then the upstream and downstream zones were heated up at the same time to 200° and 850 °C separately for 30 min and held for 10 min. The atmosphere during the whole heating process was 80 sccm Ar. 1L ReS_2_ grown in this process is triclinic and highly anisotropic, corresponding to the typical tetragonal (T) phase in TMDs with slightly distorted “diamond”‐type superlattices in two directions (*a* and *b*).

1L ReSe_2_ was grown on c‐face sapphire in atmospheric CVD. NH_4_ReO_4_ and selenium were used as precursors. The upstream zone was heated up to 400 °C at 20 °C min^–1^ and held for 10 min. Simultaneously, the downstream zone was heated at 34°C min^–1^ up to 700 °C and held for 10 min. During the growth, argon gas flow was adjusted to 80 sccm, and hydrogen gas flow was set at 1–5 sccm. Selenium vapor was carried by argon and hydrogen gas onto the c‐face sapphire substrate. Both zones cooled down naturally right at the completion of the heating program.

### (S)TEM Specimen Preparation

The CVD‐grown ReS_2_ and ReSe_2_ were transferred on TEM grids by polymethyl methacrylate (PMMA)‐assisted method. Under the rotation speed of 3000 revolutions per minute for 50 s, the c‐face sapphire substrate was covered by a thin layer of PMMA by spin coating. After being immersed in 75 °C deionized water for 2 h, the 1L ReS_2_ detached from the substrate and transferred onto a TEM grid or Protochips Fusion chip. Then the grid/chip was dried up at ambient temperature, and PMMA residue was removed by acetone vapor as the last step.

### (S)TEM Characterizations

A JEM‐ARM200F transmission electron microscope (TEM) was operated with a 60 kV accelerating voltage on monolayer ReS_2_ to avoid beam damage. The TEM has a Corrected Electron Optical Systems (CEOS) probe spherical (Cs) aberration corrector for atomic resolution in STEM mode. The electron beam current was ≈13.5 µA, and the vacuum was kept ≈1.3 × 10^−7^ mbar. During the record of the high‐angle annular dark‐field (HAADF) images, a 1.5 Å beam size 120 mm camera length was applied to STEM. The acquisition time of HAADF images was 19 µs per pixel with images formed by 512 × 512 pixels. The CL aperture during image capture was 40 µm and the collection angle was 45 to 180 mrad, to obtain the continuous atomic images with high resolution and proper contrast. The iDPC‐STEM images were acquired under a Cs‐corrected STEM (FEI Spectra 300) at 300 keV with a convergence angle of 17.4 mrad. The beam current was 20 pA, and the camera length was 145 mm, while the collection angle was set as 35–200 mrad. During imaging, the dwell time was 10 µs per pixel with 1024 × 1024 pixels each frame. Wiener filtering was applied on HAADF and iDPC images for the reduction of noises.

### In situ STEM on Phase Transition

Under high beam intensity of 0.3 pA nm^−2^, the continuous STEM electron beam scanning could easily trigger the S or Se defects in the exposed area without generating extended defects within 2–3 min. S or Se defects provided in‐plane mechanical loading with proper compressive stress for 1L ReS_2_ and ReSe_2_ to transform from T’’ phase to the T’/T phase. Real‐time movement of atoms could be recorded by the in situ STEM serial capture (13–20 s each frame) for phase and strain analysis.

### In situ TEM‐STM for Conductivity Measurement

A Nanofactory in situ STM‐TEM holder with JEOL 2100F TEM with 200kV accelerating voltage was applied for phase transition in TEM mode. The beam intensity was 7.5 pA nm^−2^ to avoid damage to the sample and create transformation to the T’/T phase. Keithley 2400 with a LabVIEW program was used for *I*‐*V* data collection. Using electrochemical corrosion under a solution of 1 m NaOH at a 6 V bias, W tips could be made with a few nanometers of diameter on the top. The tip was further cleaned with ethanol and deionized water. Connected with the Nanofactory holder, piezoelectric‐driven fine control drove W tips to move precisely in 3D space (maximum range: ±14 µm; minimum step: 2 pm).

### Strain Analysis on TEM Images

According to von Mises strain distributions, for the monolayer ReS_2_ sample, the strain could be separated into two parts, including the dilatational component and the shear component. Local strain hydrostatic invariant and local shear strain invariant was used to describe the dilatational and shear component separately in order to quantify the strain distribution in a symmetric atomic structure (T phase) with the known reference state. Therefore, the dilatational atomistic local strain in 2D materials could be expressed as:

(1) $$\eta_{m}^{2D}=\frac{1}{3}Tr\eta =\frac{1}{4}\left(d_{0}^{-1}TrM_{i}-2\right)$$

and the share invariant is:

(2) $$\eta_{s}^{2D}=\sqrt{\frac{1}{2}Tr{\left(\eta -\eta_{m}I\right)}^{2}}=d_{0}^{-1}\sqrt{\frac{1}{4}Tr{\left(M_{i}-\frac{TrM_{i}}{2}I\right)}^{2}}$$

For each neighbor atom *j* of atom *i*, *M_i_* is the sum of the cross product of the transformation matrix *J* and its transposed matrix. The six closest neighbor atoms are regarded as *j* for the strain calculation in every single atom *i* of 1T phase ReS_2_ and *d*_0_ is a constant depending on the average distance of two atoms in the symmetric reference material. The GPA strain analysis^[^ ^27^ ^]^ (on high‐resolution HAADF images) was performed with the reflexes (in reciprocal space) perpendicular to the *a* and *b*‐axis as the two bases, respectively. The GPA analysis resolution was set as 0.3–0.4 nm, and the smoothing factor was set as 7.0.

### Density Functional Theory Calculations

DFT calculations were performed using the generalized gradient approximation for the exchange‐correlation potential with a plane‐wave basis and the projector augmented wave method as implemented in the Vienna ab‐initio simulation package (VASP).^[^ ^28^, ^29^, ^30^ ^]^ The energy cut‐off for the plane wave was set to 700 eV for structural relaxation and 500 eV for electronic structure calculations, and the *k*‐point sampling of the first Brillouin zone is 7 × 7 × 1. In the energy comparison of the T phase with the T’ and T’’ phases, a 2 × 2 × 1 supercell was used to ensure the equal number of atoms in the unit/supercells of those three phases. A *k*‐mesh of 7 × 7 × 1, equivalently to a 14 × 14 × 1 *k*‐mech for the T phase unit‐cell, was used for all those three phases. The vacuum layers of all phases are >15 Å. The structures were fully relaxed until the residual force per atom was less than 0.001 eV Å^−1^. In structural relaxation and electronic property calculations, dispersion correction was considered in the optB86b functional for the exchange potential.^[^ ^31^ ^]^ Uniaxial strain along *a*‐direction is achieved by fixing the lattice constant in the *b*‐direction of the freestanding T’’ phase and changing the lattice in *a*‐direction for a fixed area relaxation. Charge doping was realized with the ionic potential method^[^ ^32^ ^]^ to ensure the doping electrons were distrusted around the doped atoms and kept the neutrality of the supercell. For electron/hole doping on S (Re) atoms, electrons/holes were removed from a 1s (2s) core level of S (Re).

### Estimation of Defect Formation Energy

The formation energy of defects is defined as *E*_form_ =  *E*_defect_ − *E*_pure_ + *nμ*_removed_, where *μ*_removed_ is the chemical potential of the removed atoms to form a defect. Chemical potentials of Re and S in ReS_2_ fulfilled the equation μRe+2μS=μRe*+2μS*+ΔHReS2, where μRe∗ is the chemical potential of Re in the bulk form,$\mu_{S}^{*}$ is the chemical potential of S in the *α*‐phase crystal form and ΔHReS2 is the formation energy of ReS_2_. When S is rich( $\mu_{S}=\mu_{S}^{*})$, $\mu_{\mathrm{removed}}=\mu_{S}^{*}$. When Re is rich ( μRe=μRe*),μremoved=μS*+ΔHReS2/2.

### Evaluating the Displacement Threshold Energy *T*_d_

This simulation was implemented using a self‐developed tool aBEST (https://gitee.com/jigroupruc/aBEST). The simulation process consisted of canonical ensemble and microcanonical ensemble molecular dynamics simulation. The energy cut‐off for the plane wave was set to 200 eV, and the *k*‐point sampling is 1 × 1 × 1 in 4 × 4 × 1 supercell for all ab‐initio molecular dynamics methods(AIMD).^[^ ^33^, ^34^ ^]^ Canonical ensemble molecular dynamics simulation was performed at 300 K for 3 ps with a time step of 1 fs to simulate a thermal vibration environment. Microcanonical ensemble simulation for 0.8 ps with a time step of 1 fs and traced the trajectory of the target S atom to judge whether the atom was out of the lattice structure. The criterion for the displacement of the target atom was set to 5 Å. A binary search method was used to obtain the range of the transferred kinetic energy until the velocity resolution was <0.001 A fs^−1^ (*T*_d_ ≈0.1 eV).

### Estimation of Electron Beam Threshold Kinetic Energy

Threshold kinetic energy *E*_e_ is described as the minimum electron beam kinetic energy required to knock out the target atom.^[^ ^35^ ^]^ As the cross‐section derived by the McKinley–Feshbach formalism^[^ ^35^, ^36^ ^]^ was equal to zero, *E*_e_ could be evaluated.

## Conflict of Interest

The authors declare no conflict of interest.

## Author Contributions

F.Z., D.P.G., L.H., and L.W.W. contributed equally to this work. J.Z., W.J., T.H.L supervised and led the research. L.H., C.S.L., and T.H.L performed the sample synthesis and characterizations. F.Z, L.W.W, Y.C., N.W., S.P.L, and J.Z performed TEM/STEM experiments and analysis. D.P.G., C.W., and W. J. performed DFT calculations and analysis. J.Z, T.H.L, W.J., F.Z., D.P.G. co‐wrote the manuscript. All authors read and approved the final manuscript.

## Supporting information

Supporting Information

## Data Availability

The data that support the findings of this study are available from the corresponding author upon reasonable request.
