# Supplementary material for: Sub‐Nanometer Electron Beam Phase Patterning in 2D Materials
Source: Adv Sci (Weinh). 2022 Jun 16;9(23):2200702. doi: 10.1002/advs.202200702 (PMC9376820; doi:10.1002/advs.202200702)
Supplement: Supplementary file 1 — Supporting Information [file ADVS-9-2200702-s001.pdf]

ADVANCED SCIENCE
Open Access

Supporting Information

for *Adv. Sci.*, DOI 10.1002/advs.202200702

Sub-Nanometer Electron Beam Phase Patterning in 2D Materials

Fangyuan Zheng, Deping Guo, Lingli Huang, Lok Wing Wong, Xin Chen, Cong Wang, Yuan Cai, Ning Wang, Chun-Sing Lee, Shu Ping Lau, Thuc Hue Ly, Wei Ji* and Jiong Zhao**

Supporting Information

Sub-nanometre electron beam phase patterning in two-dimensional materials

Fangyuan Zheng[1+], Deping Guo[2+], Lingli Huang[3+], Lok Wing Wong[1+], Xin Chen[3], Cong Wang[2], Yuan Cai[4], Ning Wang[4], Chun-Sing Lee[3], Shu Ping Lau[1], Thuc Hue Ly[3*], Wei Ji[2*], Jiong Zhao[1*]

[1] Department of Applied Physics, The Hong Kong Polytechnic University, Kowloon, Hong Kong, China & Polytechnic University of Hong Kong Shenzhen Research Institute, Shenzhen, China.

[2] Beijing Key Laboratory of Optoelectronic Functional Materials & Micro-nano Devices, Department of Physics, Renmin University of China, Beijing, 100872, China

[3] Department of Chemistry and Center of Super-Diamond & Advanced Films (COSDAF), City University of Hong Kong, Kowloon, Hong Kong, China & City University of Hong Kong Shenzhen Research Institute, Shenzhen, China.

[4] Department of Physics, Hong Kong University of Science and Technology, Clear water bay, Hong Kong, China.

*email: jiongzhao@polyu.edu.hk (J.Z.), wji@ruc.edu.cn (W.J.), thuchly@cityu.edu.hk (T.H.L.)

[+]F.Z., D.P.G., L.H and L.W.W. contributed equally in this work.

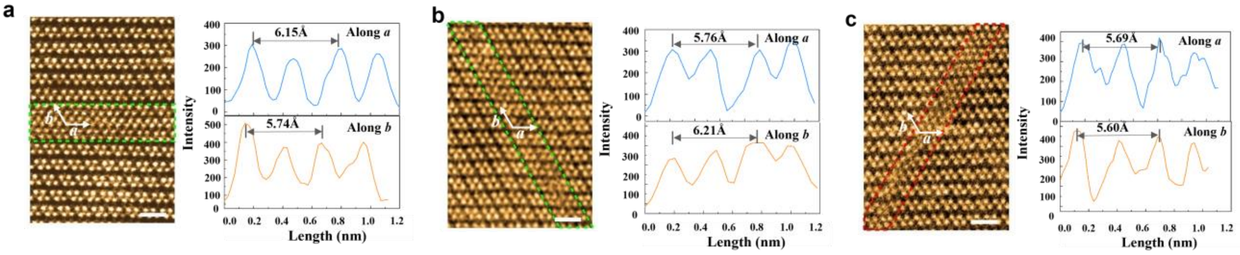

Figure S1. The measurement of lattice spacing after e$^-$ beam scanning along a) ***a*** direction, b) ***b*** direction and c) ***a+b*** direction. Scale bar =1nm.

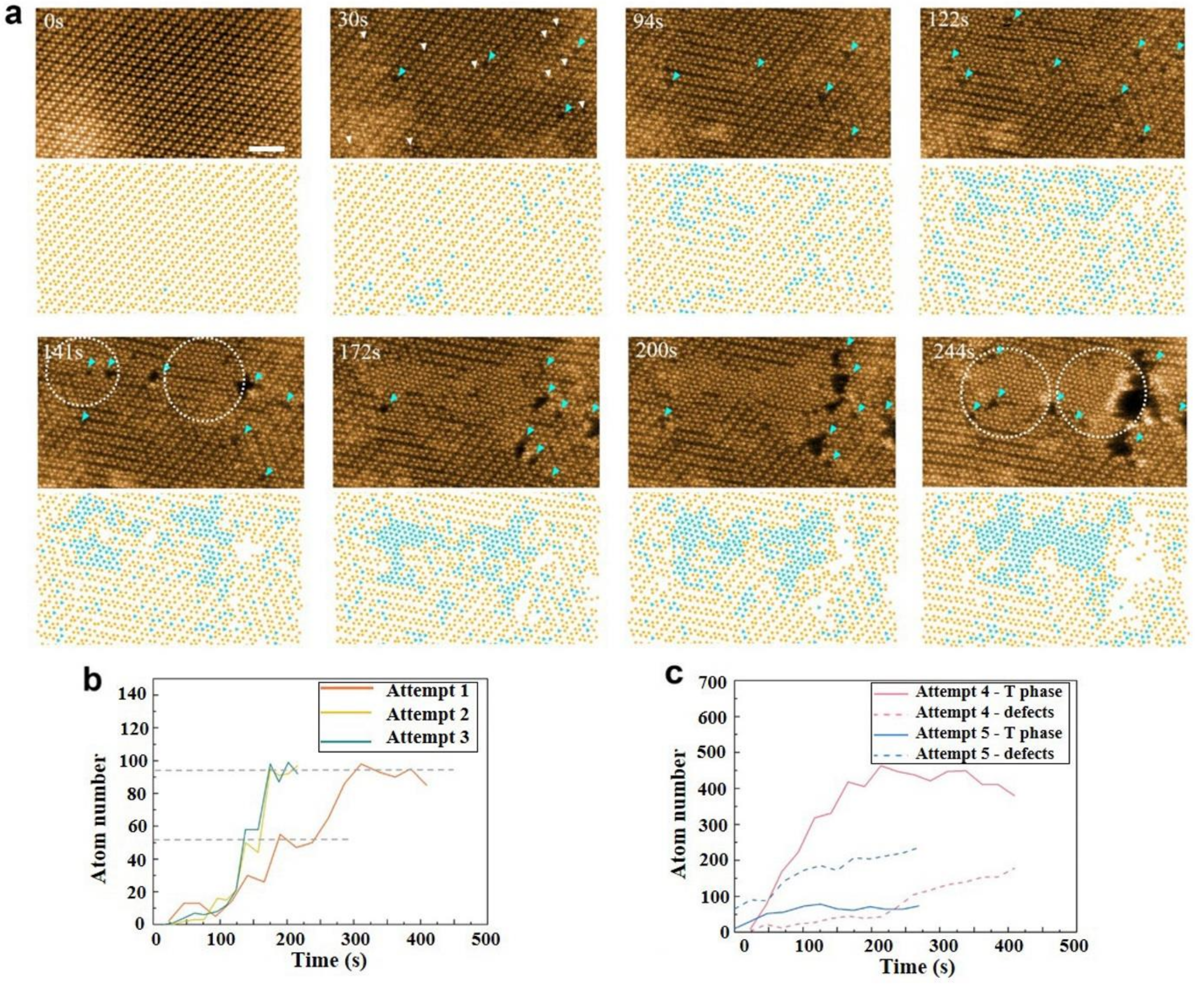

Figure S2. The 2D phase patterning in 1L ReS_2. a) Serial STEM HAADF (up) and extracted atomic position series (down) during 2D phase patterning. In HAADF images, S and Re atomic defects are highlighted by white and blue triangles, respectively. Two clusters of new T phase zones are marked by white dashed circles. In extracted atomic scheme series, yellow dots stand for T” phase, blue dots stand for T phase. Scale bar = 1.5 nm. b) Time evolution for number of T phase Re atoms in each T phase nucleus during e^- beam patterning. c) Statistics on Re atoms in new T phase and Re defects during 2D e^- beam 2D exposure in a surface area. Red lines stand for Re defect-poor case, blue lines stand for Re defect-rich case. Less Re defects promote the formation of T phase.

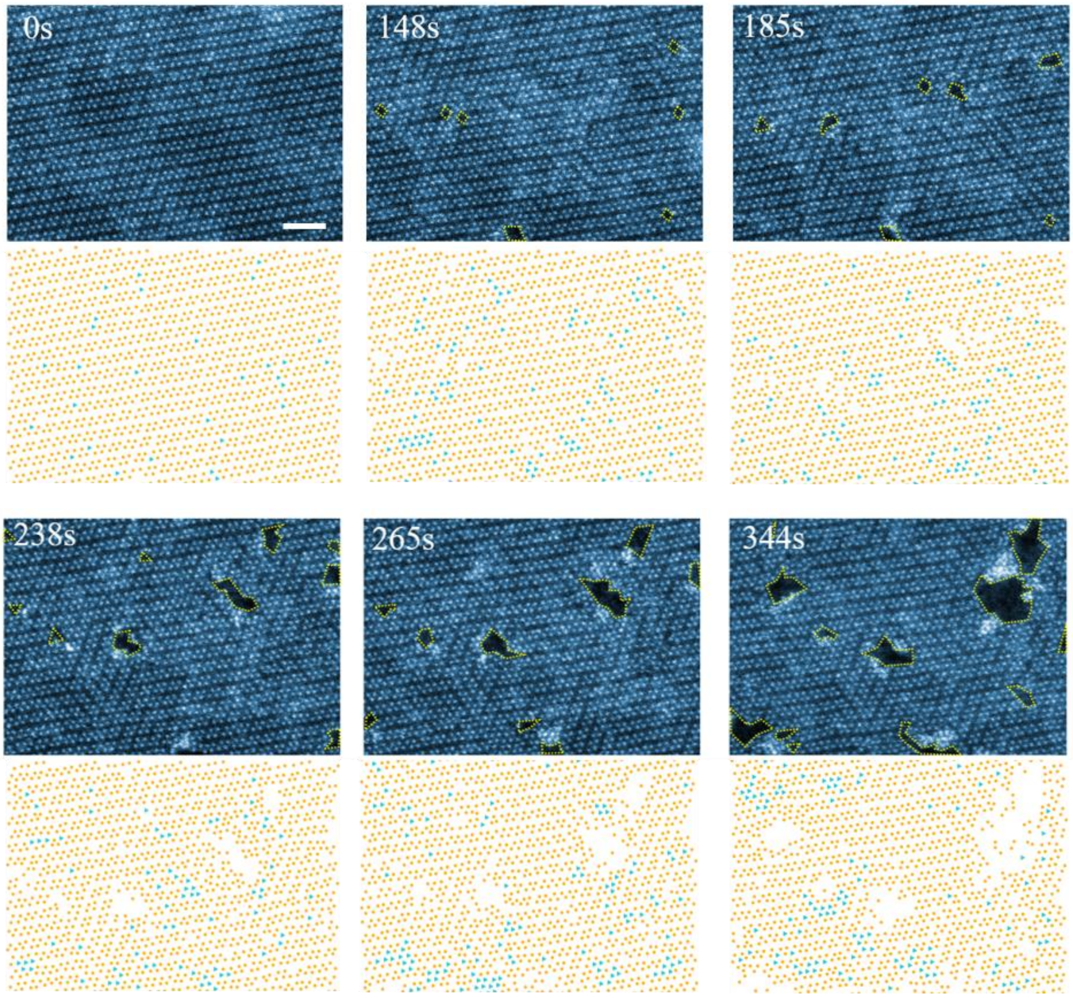

Figure S3. A serial STEM HAADF images (up) and extracted Re atomic position (down) for e^- beam exposure on 1L ReS_2 when Re vacancies are rich. Re vacancies are unfavorable for the T phase formation. Scale bar = 2 nm.

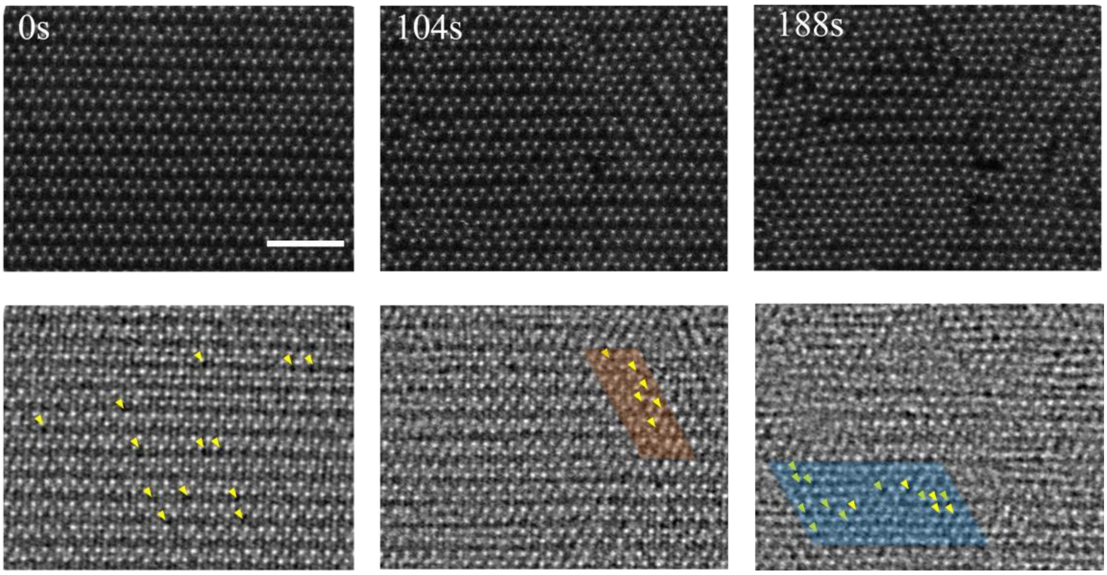

Figure S4. HAADF(up) and iDPC(down) images of phase patterning on monolayer ReS_2. S vacancies are marked by yellow arrows in T’’(0s), T’(orange area) and T (blue area) phases. Scale bar=2nm.

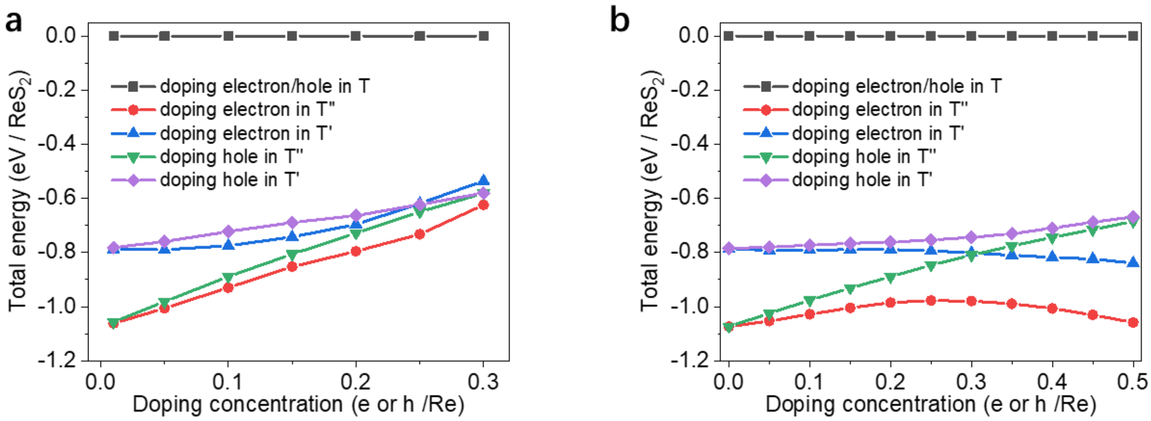

Figure S5. Influence of electron/hole doping on stability of those three phases. a) Relationship of total energies and electron/hole doping on S atoms in 1L ReS_2. b) Relationship of total energies and electron/hole doping on Re atoms in 1L ReS_2. The total energy of the T phase is set to zero (black square). Doping electron in T' and T" phase is represented by red circles and blue triangles, respectively. T' and T" phase doped hole is represented by green triangles and purple diamond, respectively.

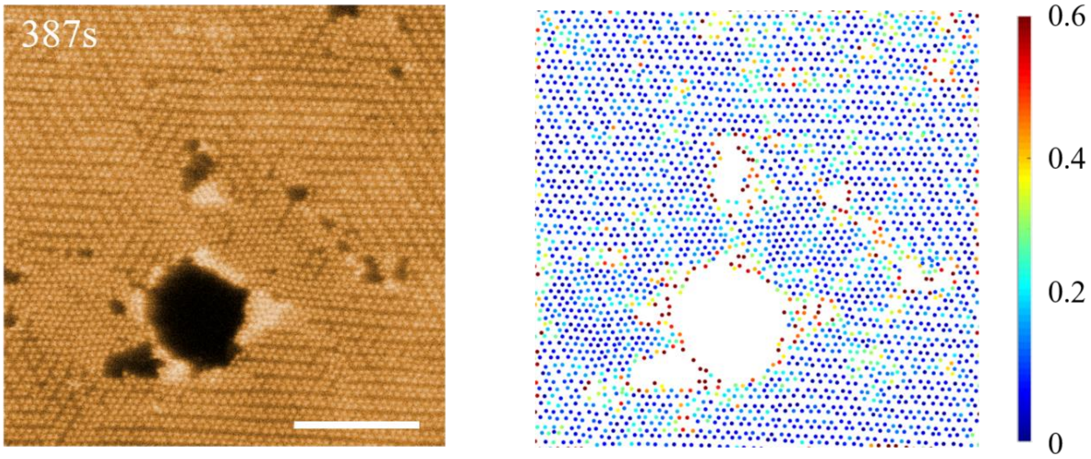

Figure S6. Shear strain mapping in 1L ReS_2. STEM HAADF image (Left) and the shear strain invariant mapping (Right) on a mixed phase (T'' and T phases) area. Scale bar = 5nm.

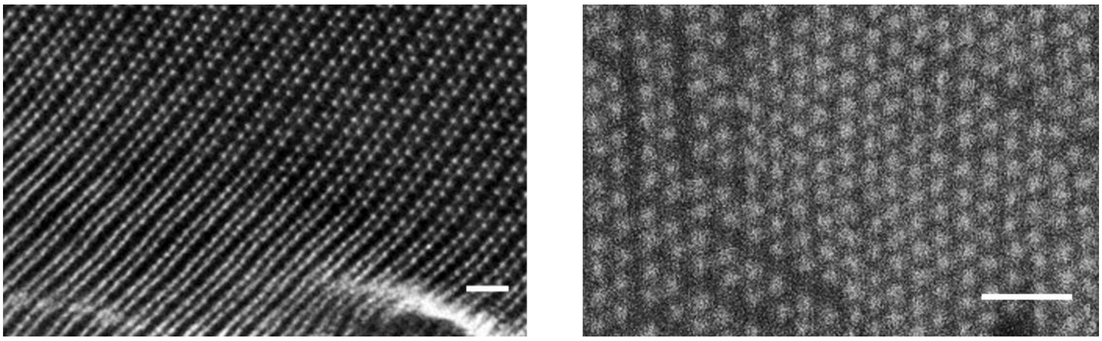

Figure S7. STEM-HAADF image for comparison of out-of-plane bending (left) and transformed T phase structure (right). Scale bar =1nm.

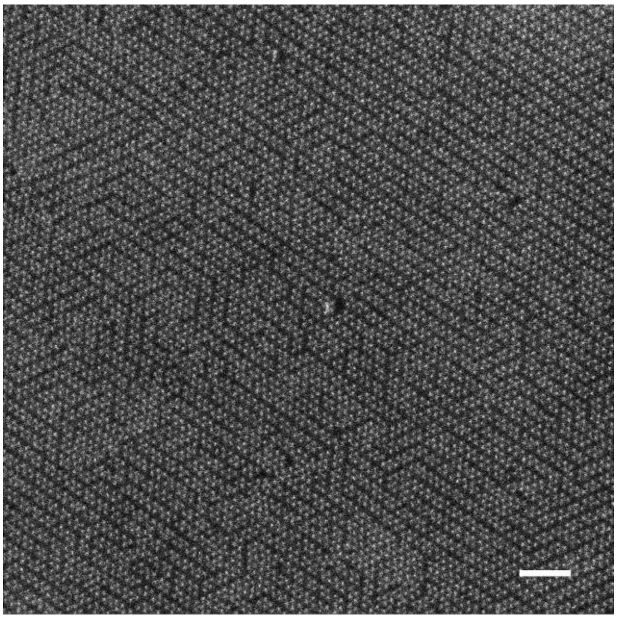

Figure S8. 2D Phase patterning on 1L ReS_2 leading to mixed phases. Scale bar =2nm.

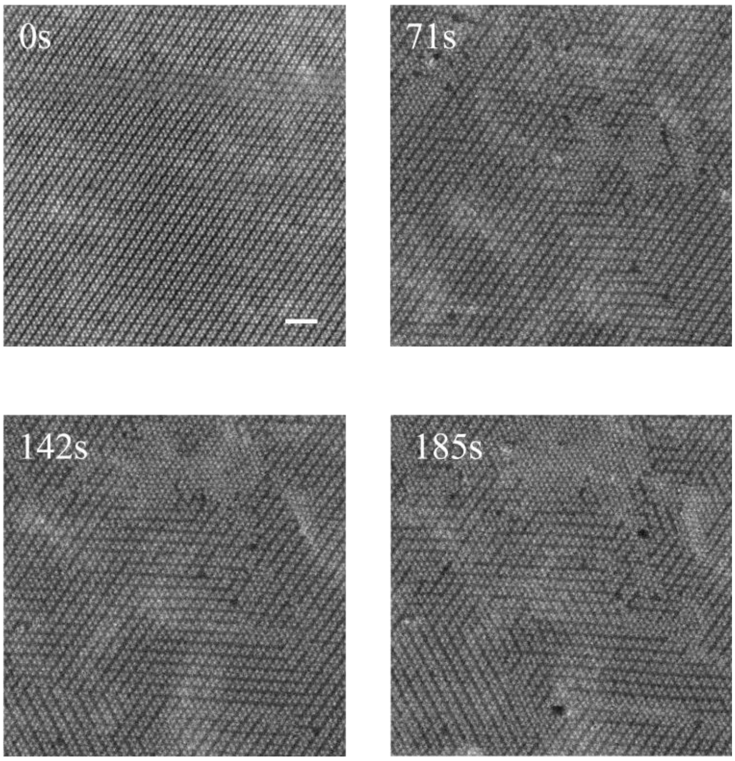

Figure S9. Phase patterning on 2D ReS_2 with big Re nanoscale poles avoided. Scale bar =2nm.

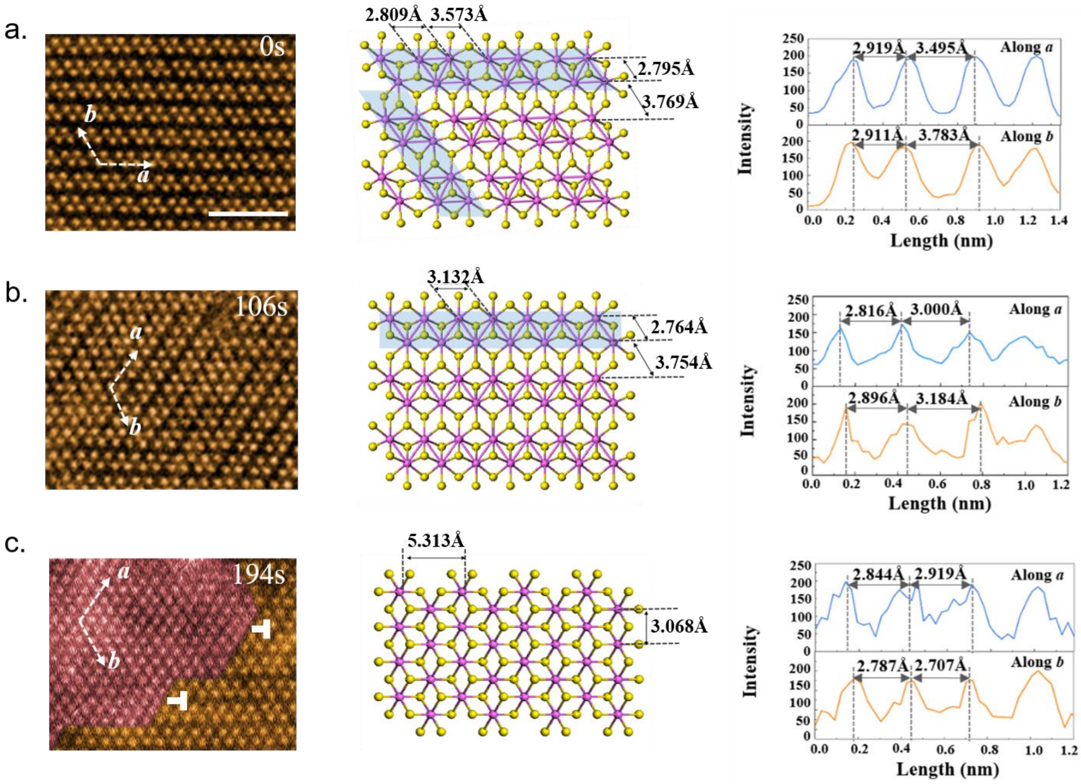

Figure S10. STEM HAADF images (left), atomic models by DFT (middle) and measured lattice constants (right) of a) T” phase, b) T’ phase, and c) T phase 1L ReS_2. The lattice constants measured in right column correspond to ***a*** and ***b*** directions labeled in the left column. In left column, the transformed T phase is highlighted by red color. Dislocations are marked by white wedges. Scale bar = 2 nm.

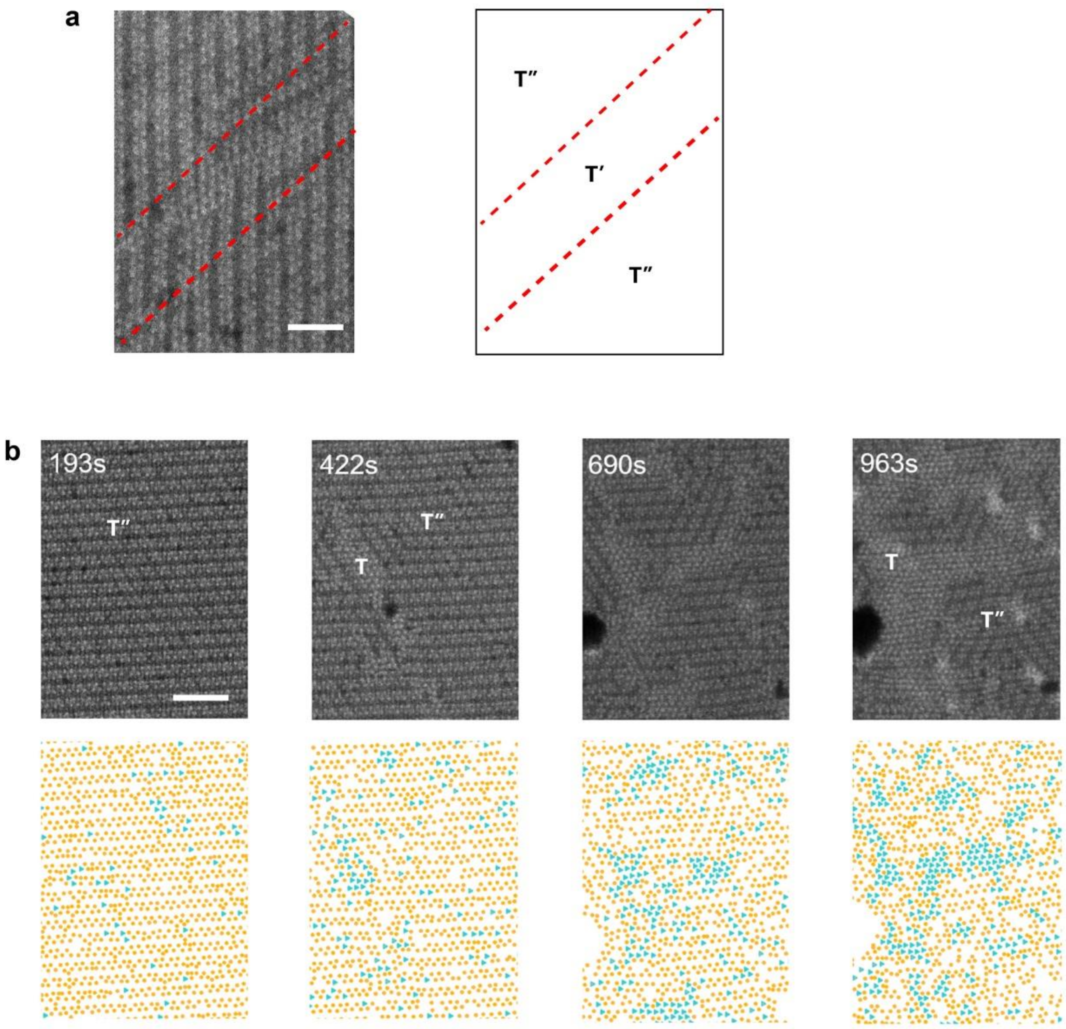

Figure S11. 1D and 2D phase patterning in 1L $ReSe_2$. a) The 1D e^- beam exposure (red dashed box) on 1L $ReSe_2$. Scale bar =1 nm. b) (upper)The serial STEM images during 2D e^- beam exposure on 1L ReS_2. (lower) The Re atomic maps corresponding to upper row. Green: T phase, yellow: T" phase. Scale bar = 2 nm.

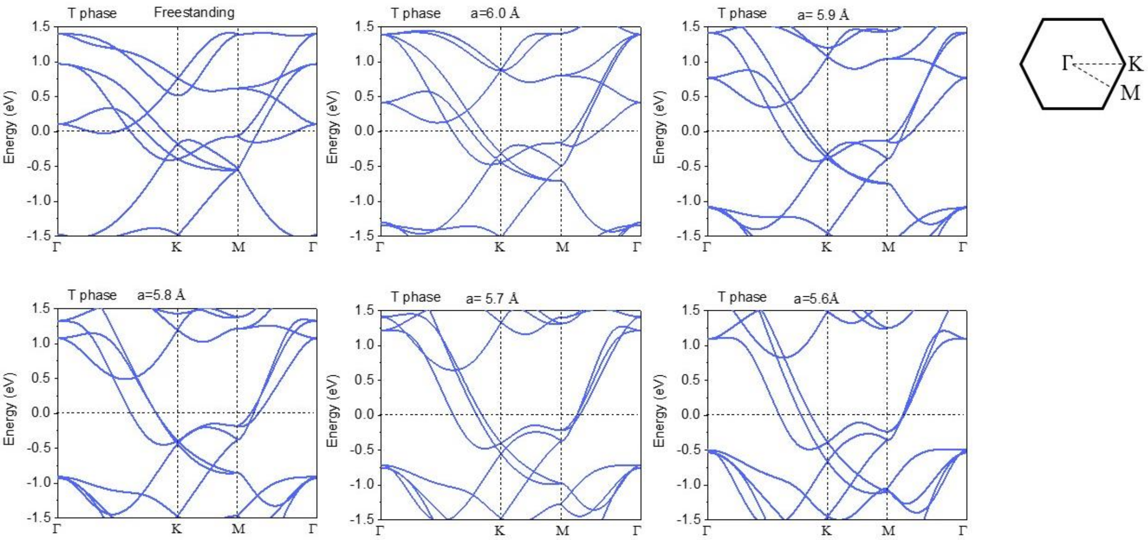

Figure S12. DFT calculated band structures for T phase (ground state and different strained states) 1L ReS_2.

S vacancy position	E_{form}(eV)	T_d(eV)	E_e(keV)
Single-1	0.13~3.35	7.13(±1.34)	95.2(±16.8)
Single-2	-0.14~3.09	7.02(±1.24)	94.0(±15.5)
Single-3	-0.99~2.25	5.88(±1.04)	79.7(±13.3)
Single-4	-0.54~2.69	6.49(±1.46)	87.3(±18.5)
Double-3,1	-0.07~3.16	7.46(±0.22)	99.3(±2.7)
Double-3,2	-0.29~2.94	7.80(±1.41)	103.4(±17.4)
Double-3,4	-0.43~2.80	6.91(±1.89)	92.6(±23.8)
Double-3,5	-0.90~2.33	6.81(±0.42)	91.3(±5.3)
Double-3,6	-0.95~2.28	6.29(±1.07)	84.8(±13.7)
Double-3,a	-0.53~2.69	>10	>150
Double-3,b	-0.03~3.20	>10	>150
Double-3,c	-0.18~3.05	9.47(±0.37)	123.4(±4.4)
Triple-3,5,4	-0.61~2.62	6.91(±2.76)	92.6(±35.2)

Table S1. S atomic vacancies (corresponding to Figure S2) formation energies (E_{form}), the displacement threshold energy (T_d) and electron beam threshold kinetic energy (E_e) in 1L ReS_2 by DFT calculation.
